# Online options for future conferences will have an important positive impact for Early Career Researchers in pediatric pain

**DOI:** 10.1002/pne2.12044

**Published:** 2020-12-10

**Authors:** Caroline Hartley

**Affiliations:** ^1^ Department of Paediatrics University of Oxford Oxford UK

**Keywords:** COVID‐19, early career researcher, virtual conference

## Abstract

The extent to which the COVID‐19 pandemic will have a negative impact on Early Career Researchers (ECRs) within the field of pediatric pain and in general is still unclear, but it is likely to be far reaching and may disproportionality affect women. Yet, there is also great opportunity to take advantage of the rapid adaptions to working life that we have all undertaken during the pandemic. In particular, continuing to have online options for conference attendance I think will have a positive impact on ECRs, and in particular female ECRs, now and in the future. Moreover, the pediatric pain research community is relatively small and highly international; by enabling wider participation in our conferences we will diversify our research output and expedite our aim of providing better treatment of pediatric pain.

The disruption the COVID‐19 pandemic has caused to research activities will be varied depending on area of research, how easy it is to resume the research once lockdowns and restrictions are increasingly eased, and the country in which the research is conducted. The pediatric pain research community is highly diverse, and we will have all been affected in different ways, with some having to stop laboratory studies, some being called back to clinical work, and some facing the challenges of how to adapt research studies to maintain follow‐up of participants. Nevertheless, it is likely that the disruption caused by the pandemic will have a greater effect on Early Career Researchers (ECRs) such as PhD students, Postdoctoral Researchers and new Principal Investigators, who often focus on a smaller number of projects compared to more senior colleagues, and who are still establishing themselves in the competitive research environment.^1^, ^2^ The hiring freezes now in place across many institutions will certainly affect those coming to the end of their current posts, and this effect will be concentrated on those at the beginning of their careers. The large impact that COVID‐19 will have on the finances of universities, research organizations and charities may mean that grant income and research jobs are more difficult to secure for a number of years to come. Moreover, increased stress and anxiety relating to work and the personal wellbeing of family and friends will negatively impact mental health,^2^, ^3^ and ECRs are more likely to be caring for young children, compared with more senior colleagues, which may additionally impact on productivity at a critical time of their career and may have a disproportionate effect on women.^4^, ^5^, ^6^

All of this paints a bleak picture for ECRs, both within and outside the pediatric pain community. However, as we now tentatively look forward to the future, it is important to consider what we can learn from the pandemic that could instead have a positive effect on ECRs and on our field of research. I believe one area which could particularly benefit from adaptions we have made during the pandemic is conference attendance. Presenting work at a conference is important for the career advancement of ECRs – they become more widely recognized by the research community leading to more citations, future grant income, and networking opportunities, and it allows them to share ideas and keep up‐to‐date with advances in the field. However, although little discussed, whilst presenting at and attending conferences has many important benefits, it can also lead to personal dilemmas for ECRs, particularly those with young children. Should/can I leave my children to go to an international conference? How many times a year do I feel that I can do this? Is my partner/someone else able to take on the burden of full‐caring responsibility for several days, and how does this fit with their own work commitments?

The extent to which conferences have an impact on work‐life balance when you have children I did not fully comprehend until I had a child of my own. On grant and career forms we are asked if we want to disclose career breaks such as maternity leave, and these are certainly important for understanding interruptions individuals may have in publishing records. However, even after I returned from maternity leave, I found that I would struggle (and still do) with going away for a meeting. Whilst my son is very young, I personally would not wish to travel to a conference which is a long flight away. This undoubtedly means that I will miss out on career opportunities. Moreover, for the conferences I have been to, particularly whilst still breastfeeding, I would often feel guilty about being away from my son, which was amplified with conferences clustered together during the summer months. The balance between attending conferences, and all the benefits we can get from that, and looking after children, will always be a difficult one. We will all navigate these things in different ways to find the best approach for us and our families. However, I believe the changes we have seen during the pandemic could help alleviate some of these problems.

The rapid transition to video conferencing that we have all seen over the past few months has marked a fundamental shift in the way we collaborate and attend conferences. In the area of pediatric pain, a number of conferences have been delayed, including the International Association of the Study of Pain (IASP) World Congress on Pain and the International Symposium on Pediatric Pain (ISPP). In place of these face‐to‐face meetings, a wealth of online conferences have been initiated, including the European Pain Federation EFIC Pain Forum Virtual Meeting (September 2020) and Virtual Pain Education Summit (November 2020), and the IASP Virtual Series on Pain and Expo which is running across a six‐ month period from September 2020 – March 2021 to enable increased accessibility and engagement. The IASP Pain Research Forum, which previously held webinars roughly every three months has been hosting them every week since May in an effort to ‘keep the pain research community connected during the COVID‐19 pandemic’.^7^

Continuing to use and further develop this technology to allow for the option of virtual conference attendance at future meetings I believe has the great potential to have an important positive impact on ECRs, allowing them to advance their careers, and helping to alleviate the anxiety, disproportionately affecting women, of attending conferences whilst parenting young children. The greater flexibility offered by providing recordings of talks online will ensure that ECRs can keep up‐to‐date with new advances in their field whilst fitting them around other commitments, including childcare. A shift to virtual conference participation precipitated by the COVID‐19 pandemic will also benefit those who are unable to attend conferences due to disability, caring responsibilities for adults, restricted funding, or for myriad other reasons.^8^ The option for virtual participation will increase diversity and allow for greater involvement of those from low‐ and middle‐income countries, maximizing research creativity and output. Pediatric pain is an international problem, and this is also reflected in the diverse international community of researchers in our field. In recent years the pain community has endeavored to reflect this by holding conferences (IASP World Congress on Pain and ISPP) across different continents, an approach which is truly commendable. However, many will still not be able to attend conferences in person or may only be able to do so periodically when the conference falls in an area of the world closer to them (given the costs and time commitments). The option to attend and present virtually at conferences going forward could truly empower pediatric pain research as a worldwide initiative. An excellent example of this is given by the Pain in Child Health (PICH) research training program. Starting in 2002, this highly successful international program aims to train the next generation of leading pediatric pain researchers, in part through monthly webinars and cross‐institution mentorship.^9^, ^10^

My hope is that conference organizers will consider the benefits of offering online participation, in addition to in person attendance, as they begin to organize meetings for the coming years. Whilst this will present additional organizational challenges, through the pandemic we have seen how quickly we are able to adapt to overcome such challenges. To help facilitate this further, funding bodies could provide additional sponsorship for conferences where online participation is offered. Some may be concerned that offering online participation may lead many researchers to select this option, altering the dynamics for those who choose to attend. Yet as it is difficult to replicate all aspects of conferences online, in particular networking, I believe those who can attend are still likely to attend. However, allowing for online attendance we will broaden the reach of the conference and diversify our research outputs. Moreover, the next few years, where some may be wary of travelling or have lower grant resources to enable them to do so, provides the ideal opportunity to trial and evaluate this type of conference attendance. Changes in culture can be galvanized during extraordinary times; whilst COVID‐19 will continue to have an impact on our work for the foreseeable future, we must use this opportunity to reflect on the changes we have made to the way we work, and the positive impact that these might have if we sustain them beyond the course of the pandemic.
